# Dynamic Obstacle Avoidance for USVs Using Cross-Domain Deep Reinforcement Learning and Neural Network Model Predictive Controller

**DOI:** 10.3390/s23073572

**Published:** 2023-03-29

**Authors:** Jianwen Li, Jalil Chavez-Galaviz, Kamyar Azizzadenesheli, Nina Mahmoudian

**Affiliations:** 1 The School of Mechanical Engineering, Purdue University, West Lafayette, IN 47907, USA; 2 Nvidia Corporation, Santa Clara, CA 95051, USA

**Keywords:** unmanned surface vehicle, deep reinforcement learning, collision avoidance, model predictive control

## Abstract

This work presents a framework that allows Unmanned Surface Vehicles (USVs) to avoid dynamic obstacles through initial training on an Unmanned Ground Vehicle (UGV) and cross-domain retraining on a USV. This is achieved by integrating a Deep Reinforcement Learning (DRL) agent that generates high-level control commands and leveraging a neural network based model predictive controller (NN-MPC) to reach target waypoints and reject disturbances. A Deep Q Network (DQN) utilized in this framework is trained in a ground environment using a Turtlebot robot and retrained in a water environment using the BREAM USV in the Gazebo simulator to avoid dynamic obstacles. The network is then validated in both simulation and real-world tests. The cross-domain learning largely decreases the training time (28%) and increases the obstacle avoidance performance (70 more reward points) compared to pure water domain training. This methodology shows that it is possible to leverage the data-rich and accessible ground environments to train DRL agent in data-poor and difficult-to-access marine environments. This will allow rapid and iterative agent development without further training due to the change in environment or vehicle dynamics.

## 1. Introduction

Unmanned Surface Vehicles (USVs) should be capable of detecting and avoiding marine obstacles, including other watercraft, buoys, marine life, and more. Avoiding dynamic obstacles is important for any autonomous deployment regardless of the domain to avoid harm to others and the vehicle itself. However, the unique aspects of surface water deployments present unique challenges due to the fluid environment. The viscous and damping nature of water means that rapid direction and velocity changes or stops can not be realized, creating a need for predictive-capable control. Furthermore, boats and water-capable vehicles are typically underactuated, this limits the number of enactable paths that can be followed to avoid obstacles. In international maritime shipping, these issues have been resolved through a standardized set of avoidance maneuvers between ships called COLREGS (The International Regulations for Preventing Collisions at Sea) [1] that can be used for autonomous systems. However, these regulations are focused on operation and avoidance at sea, and thus are only applicable to a subsection of the USV deployments.

Traditional motion planning algorithms exhibit some limitations in terms of reactive behavior owing to the computational effort required to replan a navigation path [2]. Moreover, one key aspect in path planning is the representation of the map (e.g., grid-based, polygon-based, …) [3]. When the scenario is substantially large, grid-based methods will suffer from imprecise obstacle representations or big memory demands [4]. On the other hand, geometric representations of the map can be more efficient in terms of memory consumption while suffering from a high computational cost when representing obstacles with complex shapes. In addition, the traditional path planning algorithms may require a fine-tuning stage of their parameters in order to adapt the algorithm to a previously unseen scenario [5].

Traditional dynamic obstacle avoidance methods typically employ forward-looking sensors to detect and predict the obstacle motion. Existing work on single-agent dynamic collision avoidance can be broadly classified into Model Predictive Control (MPC) and collision avoidance algorithms. Both methods turn fused environmental sensing inputs (Lidar, radar, images, etc.) into actionable robotic commands through trajectory changes or direct low-level vehicle actuation. Fused sensor data are utilized to provide obstacle tracks, generate an action space of trajectories, and use fuzzy logic blocks to calculate collision risks and refine the action space with respect to Time to the Closest Point of Approach (TCPA) [6]. In the ground domain, some collision avoidance approaches forgo path planning and path following entirely, instead using a particle model to generate acceleration vector motion plans for lower control levels to interpret into actuator commands [7].

Frequently used in marine settings, MPC uses a data-driven or numerical model to relate control outputs to vehicle state changes relative to environmental and programmatic constraints along with forcing from external forces and torque on the vehicle. The benefit of MPC for obstacle avoidance is the ability to take future time states into account when generating control outputs for a current state. Such MPCs have been implemented on drones [8], and on USVs using COLREGs and custom cost functions [9]. A good example of this is presented in [10], where a branching course MPC model is used to provide local collision avoidance paths. A survey of collision avoidance on USVs has been previously documented in [11].

Recent advances in computational power have enabled the application of Deep Reinforcement Learning (DRL) and Imitation Learning (IL) to achieve new capabilities beyond those provided by classical navigation. DRL enables an agent to continuously improve itself through experienced interactions with its environment and achieve human-level control [12]. DRL has been successfully employed to solve obstacle avoidance problems in different domains such as ground, air, and water. In the ground domain, a recurrent neural network and novel Local-Map Critic (LMC) architecture has been built to overcome the limited field of view (FOV) for mobile robot navigation [13]. In the air domain, Deep Deterministic Policy Gradient (DDPG) was adopted to develop a mapless reactive navigation algorithm applied to multirotor aerial robots [5]. In the water domain, as an example, a DRL-based controller using proximal policy optimization (PPO) was implemented on a small-sized supply ship model equipped with a rangefinder sensor suite in a purely synthetic environment [14]. Approaches based on IL [15] take examples of successful navigational behaviors given by other agents (such as humans) and use them to learn policies that result in behaviors that are similar to the examples. Most works use end-to-end training and achieve good results for specific vehicles in certain scenarios. However, if the vehicle dynamics change or the environment changes, the trained agent will not be able to perform well directly because retraining will be needed. Unfortunately, techniques that are based on RL typically are trained from scratch and depend on a significant amount of training experience, whereas IL based techniques require an expert-level demonstration carried out in the same kind of setting.

Therefore, it is preferable for a learning algorithm to use acquired knowledge to enhance performance on other tasks. Cross-domain transfer learning has achieved significant success in Reinforcement learning applications [16,17]. UGVs have been under development for decades [18] and are easier to deploy and control while USVs are susceptible to wind and wave disturbances and are typically larger than ground vehicles, which makes USVs difficult to deploy and train [19,20]. In this way, developing an obstacle avoidance strategy for the ground domain and then transferring that knowledge to the water domain is an ideal result. However, RL algorithms that can efficiently transmit policies from the ground domain to the water domain have not yet been reported.

**Figure 1:**
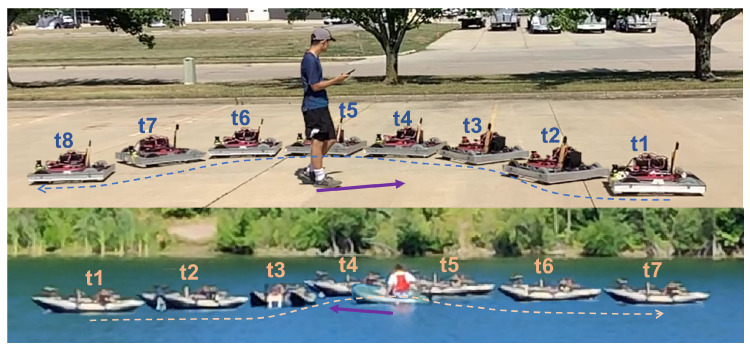
Motion lapse images of a UGV and a USV avoiding a dynamic obstacle using the same trained DRL agent. The trajectories of the vehicles are shown as dashed lines. The direction of the instantaneous velocity of the obstacles is shown as purple arrows.

This work builds on our previous work [21], where we proposed a multi-layer methodology for training a network to avoid static obstacles; then, the network was generalized across domains without further training, thus, alleviating the data sparsity problem present in the marine environment. However, it is difficult to achieve good performance with this approach when the obstacles become dynamic. This is mainly because of the limitation of the network structure and lack of further training in another domain. In this work, the research effort is focused on the development of an efficient reactive navigation algorithm able to deal with the aforementioned limitations, with particular attention to unknown scenarios with dynamic obstacles, as shown in Figure 1. In summary, the key contributions of this paper can be summarized as:

1. Building training and testing simulation environments where the moving obstacles can be integrated in random order to better train the agent and better evaluate the effectiveness of the trained agent;
2. Designing the DQN network architecture to enable the agent to avoid dynamic obstacles. Specifically, we perform min pooling on input Lidar ranges to keep high accuracy while low dimensionality. We use Long Short-Term Memory (LSTM) as a building block to process the entire sequence of data to help the agent better capture the motion of the obstacles;
3. Presenting a method that successfully enables cross-domain training and validation of a DQN across domains, moving from data-rich and accessible environments to data-poor and difficult to access environments;
4. Using a neural network to approximate the dynamics of USV system and implementing NN-MPC waypoint tracker as a low-level controller for the DRL agent to effectively avoid obstacles while rejecting disturbances that are not included during DQN training.

The remainder of this work covers the methodology utilized in DRL agent implementation, the software and hardware package utilized in real-world testing, and a description of the robotic platforms used are detailed in Section 2. The results of the test in simulated and real-world environments are presented in Section 3. Finally, the conclusions of the paper and future work are presented in Section 4.

## 2. Methodology

**Figure 2:**
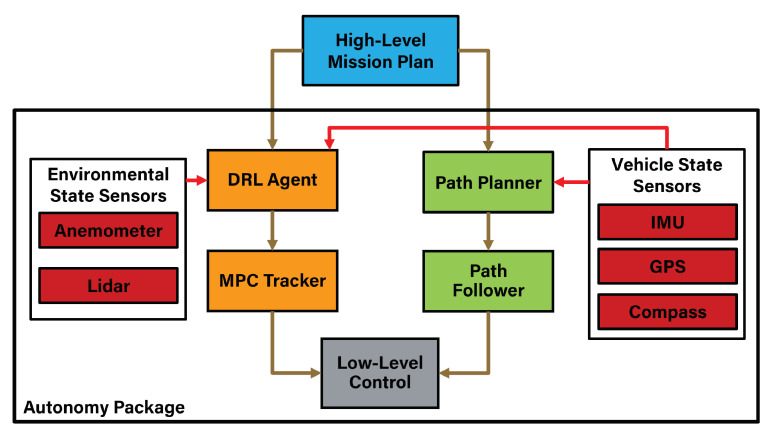
Autonomy package including all the control levels and sensors.

The autonomy package of the USV presented in this work consists of two control strategies and multiple sensors. Some of these sensors, such as IMU, GPS, and compass, monitor the vehicle’s state. In contrast, other sensors such as Lidar and anemometer enable obstacle detection and wind rejection, respectively. During a regular course, when no obstacle is in range, a path planner and ILOS follower [22] have control of the USV, guiding it through a path P of length L, which consists of a sequence of waypoints. To prevent collisions, once an obstacle is detected, a DRL agent takes over the control of the USV, generating a temporary waypoint located at $\left(N_{temp},E_{temp}\right)$. This waypoint is obtained based on the current state and the DRL agent’s prediction. Next, this temporary waypoint is passed to a neural network model predictive controller (NN-MPC) that considers the vehicle’s state and the disturbances to drive the USV through an obstacle-free trajectory. A block diagram of the proposed scheme is shown in Figure 2.

**Figure 3:**
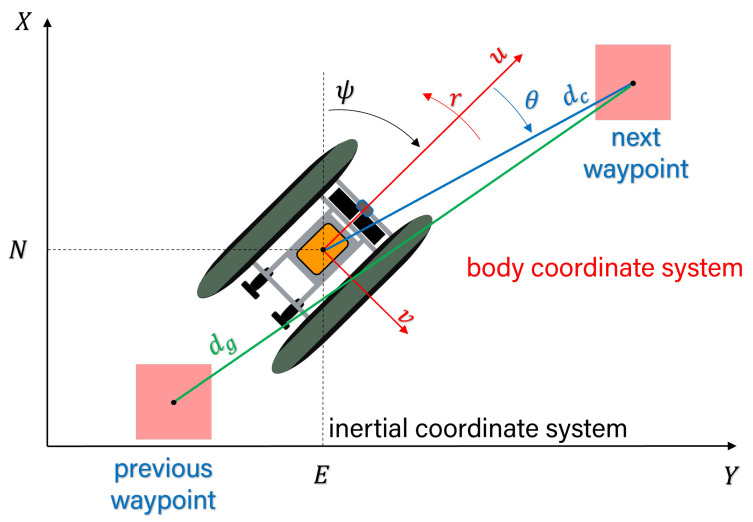
The vehicle’s state in the inertial and the body fixed frame.

To achieve precise control of the USV during obstacle avoidance, a motion model is essential. As described in [20], a 3-DOF mathematical model of a USV can be described as follows:

(1) $$\begin{array}{c} \dot{\eta }=R\left(\psi \right)v \end{array}$$

(2) $$\begin{array}{c} M\dot{v}+C\left(v\right)v+D\left(v\right)v=\tau +\tau_{w} \end{array}$$

where $\eta ={\left[N,E,\psi \right]}^{T}$ defines the USV’s pose in an inertial coordinate system as illustrated in Figure 3. The speed vector $v={\left[u,v,r\right]}^{T}$ consists of the linear velocities (u,v) in the surge and sway directions and the rotation velocity. The thrust vector τ contains the force and moment produced by the port and starboard trolling motors $u^{thrust}={\left[u^{p},u^{s}\right]}^{T}$. The wind disturbance $\tau_{w}$ is produced by the wind speed $w={\left[w^{u},w^{v}\right]}^{T}$ measured in the surge and sway directions of the USV. The matrix R(ψ) is the rotation matrix used to transform from the body fixed frame to the earth fixed frame. M, C(v), and D(v) represent the inertial mass matrix, the Coriolis centripetal force matrix, and the damping matrix, respectively.

With the 3-DOF mathematical model of the USV, we can accurately predict the motion of the vehicle in response to various inputs. However, to operate the USV in an environment with obstacles, and in presence of disturbances, we need to take into account the potential for collisions. The following sections provide detail about autonomous navigation and obstacle avoidance strategies.

### 2.1. Path Planner

Path planning for marine systems generates curves to connect waypoints produced by a high-level mission planner. In this work, during normal operation, a Dubins path planner is responsible for generating a feasible path for the USV. The Dubins path generation considers an initial and a final point in the configuration space of the vehicle. The goal is to find the shortest smooth path connecting the initial and final configurations with a curvature restriction $\frac{1}{\rho }$. This problem was first solved by [23] showing that the solution must be composed of three fundamental path segments following the sequence CCC or CSC where *C* is an arc that can be either clockwise *R* or counter-clockwise *L*, and *S* is a straight segment. This gives as result, an admissible set of possible paths, which are the building blocks of the path P. Each waypoint in P is used as a reference input for the ILOS path follower.

### 2.2. Path Follower

The process of path following involves guiding a vehicle to follow a predefined path P in space, even in the presence of unknown disturbances. In the context of ships and offshore platforms, the path following problem is often simplified to a 2-D path. A widely used approach to solve the path following problem for marine systems is the Line Of Sight (LOS) navigation due to its performance and simplicity; however, LOS guidance has some limitations when the vehicle is in presence of unknown disturbances [22,24], caused mainly by waves, wind or oceanic currents. To overcome the weaknesses of the LOS guidance law, it is necessary to include an integral action to aid in the disturbance rejection and also in the elimination of the steady-state error. This change to the LOS follower can be observed in Equation (3).

(3) $$\phi_{D}=\gamma_{P}+tan^{-1}\left(-\frac{1}{\Delta }y_{e}-\hat{\beta }\right)$$

where $\phi_{D}$ denoted the desired bearing, $\gamma_{P}$ the path tangential angle at the USV’s projected position, Δ the user-specified look-ahead distance, $y_{e}$ cross-track error, $\hat{\beta }$ the adaptive estimate of sideslip angle after integration Equation (4).

(4) $$\dot{\hat{\beta }}=\gamma_{\beta }\frac{U\Delta }{\sqrt{\Delta^{2}+{\left(y_{e}+\Delta \hat{\beta }\right)}^{2}}}y_{e},\gamma_{\beta }>0$$

where *U* is the vehicle forward velocity and $\gamma_{\beta }$ is an adaption gain that weights the integral component in the desired heading calculation.

### 2.3. DRL Agent

**Figure 4:**
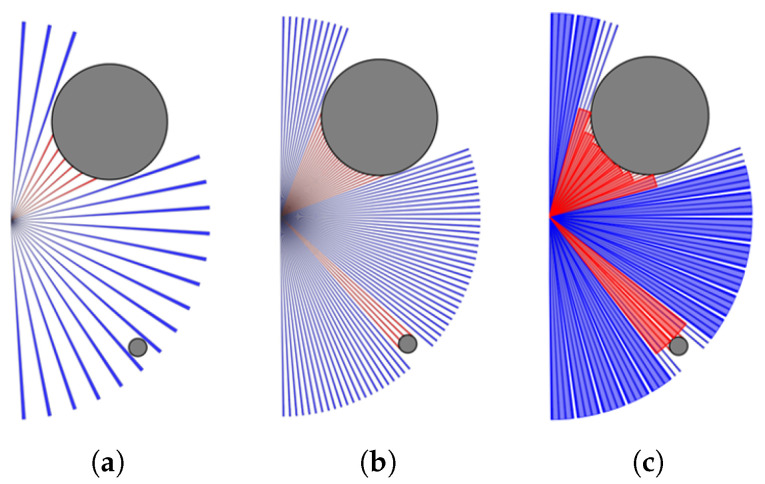
Lidar ranges visualization. Ranges in red mean obstacles are detected. (**a**) Without min pooling, the small object might not be detected with 24 samples. (**b**) The small object can be detected with 96 samples. (**c**) Using min pooling, the small object can be detected with 24 samples.

Using rangefinder sensors such as Lidar enables a relatively straightforward transition from the simulated environment to the real world. The sensor requires a small angular resolution to capture plenty of data, thus enabling the detection of small obstacles. However, if the large sensor output is fed to the neural network directly, the training may suffer from the curse of dimensionality [25]. To reduce the dimensionality while keeping the resolution, min pooling is utilized. As shown in Figure 4c, the size of the sensor output is 96, with a filter size of 4 and stride of 4. The output can be downsized to 24 samples.

**Figure 5:**
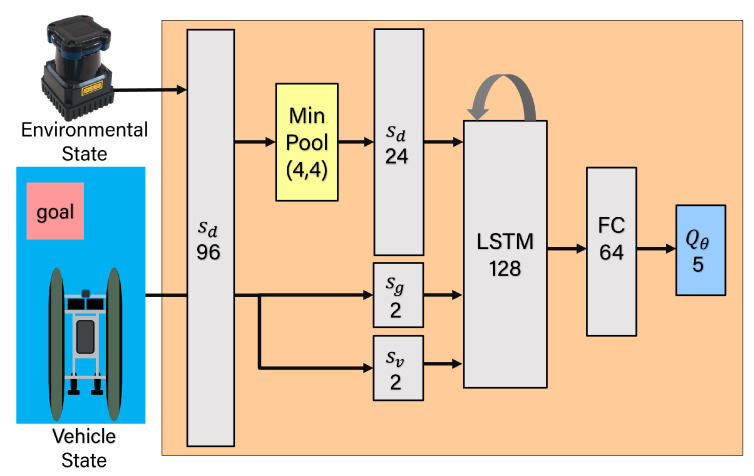
Architecture of the DQN model implemented in this work. This diagram shows the inputs ($s_{d},s_{g}$ and $s_{v}$) as well layers using as part of the overal DQN.

The neural network architecture used to generate Q-value estimation is shown in Figure 5. To capture the motion of dynamics obstacles we define a new observation space $s_{t}$ as given in Equation (5). The observation space $s_{t}$ consists of the readings of sensor $s_{d}$, relative position to the goal $s_{g}$, and the robot’s current velocity $s_{v}$. $s_{d}={\left[d_{1},d_{2},\ldots ,d_{24}\right]}^{T}$ includes the measurements from range finder sensors. The relative goal position $s_{g}={\left[g_{1},g_{2}\right]}^{T}$ contains the distance and angle with respect to the robot’s current position. The observed velocity $s_{v}={\left[v_{1},v_{2}\right]}^{T}$ includes the current translational and rotational velocity of the robot. All of the observations are normalized to [0,1] and finally, a sequence of 4 consecutive samples $\left[s_{t-3},s_{t-2},s_{t-1},s_{t}\right]$ is fed into the Long Short-Term Memory (LSTM) layer.

(5) $$s_{t}={\left[d_{1},d_{2},\ldots ,d_{24},v_{1},v_{2},g_{1},g_{2}\right]}^{T}$$

LSTM [26] is a well-known architecture for recurrent neural networks. It includes a memory unit *c*, a hidden state *h*, and three varieties of gates: an input gate *i*, a forget gate *f*, and an output gate *o*. These gates are utilized to regulate memory unit reading and writing. For each time step *t*, LSTM receives an input $s_{t}$ and the previous hidden state $h_{t-1}$, then computes gate activation, and finally updates the memory unit to $c_{t}$ and the hidden state to $h_{t}$. The computation involved is as follows:   

(6) $$\begin{array}{cc} i_{t} & =\sigma (W_{xi}s_{t}+W_{hi}h_{t-1}+b_{i}),\\ f_{t} & =\sigma (W_{xf}s_{t}+W_{hf}h_{t-1}+b_{f}),\\ c_{t} & =f_{t}⊙c_{t-1}+i_{t}⊙\tanh\left(W_{xc}s_{t}+W_{hc}h_{t-1}+b_{c}\right),\\ o_{t} & =\sigma (W_{xo}s_{t}+W_{ho}h_{t-1}+b_{o}),\\ h_{t} & =o_{t}⊙\tanh\left(c_{t}\right) \end{array}$$

where σ(x)=1/(1+exp(−x)) is a logistic sigmoid function, ⊙ denotes the point-wise product, and *W* and *b* are weights and biases for the three gates and the memory unit.

Due to LSTM’s ability to memorize long-range context information, it has been extensively exploited to address a variety of problems concerning sequential data analysis. Here we utilize LSTM to extract features from the sequence of states $s_{t}$ of the USV.

(7) $$R\left(s_{t},a_{t},s_{t+1}\right)=\left\{\begin{array}{c} +1\;(\mathrm{Goal}\;\mathrm{reward})\\ -1\;(\mathrm{Collision}\;\mathrm{penalty})\\ R_{\theta }R_{d}+R_{a}\;(\mathrm{Position}\;\mathrm{reward}) \end{array}\right.$$

$R_{\theta }$, $R_{d}$, and $R_{a}$ are the heading reward, distance reward, and action reward respectively, and are defined in Equation (8). θ is the yaw offset from the goal, $d_{c}$ is the current distance to the goal, and $d_{g}$ is the initial distance to the goal.

(8) $$R_{\theta }=0.05\left(1-\frac{2}{\pi }\left|\theta \right|\right)$$

(9) $$R_{d}=2^{\frac{d_{c}}{d_{g}}}$$

(10) $$R_{a}=\left\{\begin{array}{c} -0.01\;(\mathrm{Hard}\;\mathrm{turns})\\ +0.01\;(\mathrm{Go}\;\mathrm{straight})\\ 0\;\left(\mathrm{Others}\right) \end{array}\right.$$

To improve real-world applications, a deep neural network is used as a function approximator (Q(s,a)) to give a *Q*-value for any input state-action pair; such a deep neural network is referred to as a DQN. Thus the goal of agent training is to train the neural network to accurately approximate *Q*-values using the following updating rule:

(11) $$\begin{array}{c} Q\left(s_{t},a_{t}\right)\leftarrow \left(1-\alpha \right)Q\left(s_{t},a_{t}\right)+\alpha \left[r+\gamma \max_{a_{t+1}}Q_{target}\left(s_{t+1},a_{t+1}\right)\right] \end{array}$$

where *t* denoted the current timestep, α the learning rate, *r* recorded rewards, γ the the discount factor. During agent operation, the DQN follows the same principles as Q-Learning: it observes the state, chooses an optimal or uniform random action as dictated by ϵ-greedy policy, transitions to the next state, and receives a reward. The agent stores its interactions with the environment in an experience replay buffer. During training, these interactions—in the form of ($s_{t},a_{t},s_{t+1},R\left(s_{t},a_{t},s_{t+1}\right)$)—are randomly extracted in batches and replayed for the DQN, which predicts the Q-value of the state action pair $Q_{\theta }\left(s_{t},a_{t}\right)$. With the predicted value and a target reward (Equation (11)), Mean Squared Error (MSE) loss, and an RMSprop optimizer; the DQN model can be iteratively trained. As experience is gained, and training progresses, the DQN network improves its performance, making it capable of better selecting an optimal action that maximizes the sum of all discounted future rewards.

The larger action spaces have a cost of more complex networks and longer training time. Training time and neural network complexity can be reduced by restricting the system to a small action area. A small action space $A={\left\{a_{i}\right\}}_{i=1}^{5}$ is selected. The DQN’s action space is comprised of five discrete actions that are aimed to follow one of five different waypoints and the action is chosen by the ϵ-greedy algorithm. Though the agent has a discrete action space, using a waypoint tracker such as MPC, it can still generate a smooth path. All of these waypoints lie on a circle of radius *D* centered at the vehicle’s current position (N,E), and are separated by an angle *d*. In ground domain tests D=3 m d=0.52 rad ($30^{\circ }$). In water domain tests D=8 m and d=0.26 rad ($15^{\circ }$) because the BREAM USV [27] has a larger turning radius. By finding the adequate action $a_{i}$ we can determine the target heading $\phi_{temp}$ (see Equation (12)), and then a waypoint located at $\left(N_{temp},E_{temp}\right)$ that is given as a goal to the NN-MPC to avoid obstacles.

(12) $$\begin{array}{cc} \phi_{temp} & =\psi +\left(3-i\right)d\\ N_{temp} & =N+cos(\phi )D\\ E_{temp} & =E+sin(\phi )D \end{array}$$

**Figure 6:**
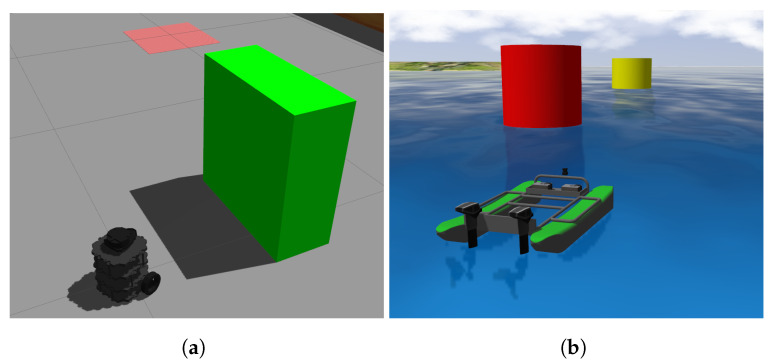
USV and UGV vehicles used in cross-domain DRL training. (**a**) Turtlebot in simulation. (**b**) BREAM in simulation.

A UGV and a USV in Figure 6 are used to train the DRL agent. Instead of directly training the agent on the USV, initial training is carried out on the UGV because the ground environment is less computational expressive to simulate, and the agent can have more interactions within same amount of time compared to the water domain due to the UGV is more agile and the environment can be more compact. Next, the policy developed from the ground domain is transferred to the water domain and is further trained.

### 2.4. NN-MPC Tracker

The DQN agent’s state used in this work does not consider disturbances; therefore, we propose a neural network model predictive controller to follow the waypoint located at $\left(N_{temp},E_{temp}\right)$ generated by the DQN agent while rejecting wind disturbances.

We used the truncated simulation error minimization approach described in [28] to train a model $N_{f}\left(\cdot ;\theta \right)$ that describes the vehicle’s motion. This approach has the advantage of including unmodelled dynamics of the system and does not require estimating individually the parameters of Equation (2). In this way, we can calculate the acceleration of the USV as follows.

(13) $$\begin{array}{c} {\hat{\dot{v}}}_{t}=N_{f}\left({\hat{v}}_{t},u_{t}^{thrust},w_{t};\theta \right)\\ {\hat{v}}_{0}=v_{0} \end{array}$$

This network estimates the linear and angular accelerations of system state ${\hat{\dot{v}}}_{t}$, given the known initial condition $v_{0}$, the current estimated state ${\hat{v}}_{t}$, the measured wind speed $w_{t}$, and the current control input $u_{t}^{trhust}$. The following equations are used to update the pose η and velocity v of the system:

(14) $$\begin{array}{c} {\hat{v}}_{t+1}={\hat{\dot{v}}}_{t}\Delta t+{\hat{v}}_{t} \end{array}$$

(15) $$\begin{array}{c} {\hat{\eta }}_{t+1}=R\left(\hat{\psi_{t}}\right){\hat{v}}_{t}\Delta t+{\hat{\eta }}_{t} \end{array}$$

MPC as a model-based technique requires an accurate way to determine the system’s state based on calculated outputs within a time horizon window *T*. To have better control over the system states, the state space of the USV is defined to include velocity, and position $x_{t}={\left[v_{t}^{T},\eta_{t}^{T}\right]}^{T}\in X$. The future relative wind velocity cannot be measured, thus we assume the wind speed is constant in the global frame during prediction. We can obtain the relative wind velocity by multiplying the initial wind speed by a transformation matrix using the predicted heading. The control output $u^{thrust}\in U$ corresponds to the thrust generated by the two trolling motors. In this way MPC is configured to solve the following optimization problem:

(16) $$\begin{array}{c} x_{1:T}^{*},u_{1:T}^{thrust*}=\underset{x_{1:T}\in X,u_{1:T}^{thrust}\in U}{arg min}\sum_{t=1}^{T}C_{t}\left(x_{t},u_{t}^{thrust}\right)\\ \mathrm{subject}\;\mathrm{to}\;x_{t+1}=f\left(x_{t},u_{t}^{thrust},w_{t}\right),x_{0}=x_{init} \end{array}$$

where the cost function is defined in terms of the augmented state vector $\alpha_{t}={\left[x_{t}^{T},{u_{t}^{thrust}}^{T}\right]}^{T}$, the augmented goal state $\alpha^{*}={\left[{x^{*}}^{T},0\right]}^{T}$, and the goal weight vector $g_{w}={\left[k_{u},k_{v},k_{r},k_{N},k_{E},k_{\psi }\right]}^{T}$ as follows:

(17) $$\begin{array}{c} C_{t}\left(x_{t},u_{t}^{thrust}\right)=\frac{1}{2}\alpha_{t}^{T}D\left(g_{w}\right)\alpha_{t}-{\left(\sqrt{g_{w}}\circ \alpha^{*}\right)}^{T}\alpha_{t} \end{array}$$

where $D(g_{w})$ is a diagonal matrix including the goal weights, and can be adjusted to achieve the desired behavior of the system, and also to penalize large control actions. With the optimization problem defined, the procedure can be repeated to obtain the optimal control output over the time horizon.

### 2.5. Field Test Implementation

**Figure 7:**
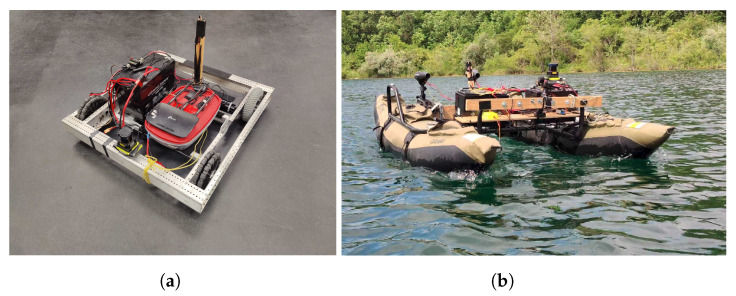
USV and UGV vehicles built for use in cross-domain DRL development. (**a**) UGV. (**b**) BREAM.

To test the ability of DRL agent to generalize across domains without additional training in real-world environments, the trained agent is implemented on two custom-made vehicles, one UGV (see Figure 7a) and one USV BREAM (see Figure 7a). To facilitate the rapid low-cost deployment of machine learning approaches across multiple domains an autonomy package has been developed. The entire package is contained within a splash-proof container that can be seamlessly integrated into the UGV or USV. The autonomy package includes two Raspberry Pi 4B and an Nvidia Jetson Nano capable of running the DQN. These computational engines are operating together in a distributed processing environment managed by the Robotic Operating System (ROS) [29]. Each processing module operates at a different level of vehicle abstraction. The package’s two Raspberry Pis operate as a frontseat-backseat duo, while the Jetson provides prediction and insight into the vehicle’s changing environment.

To provide observation space (Equation (5)) values, a Hokuyo UTM-30LX-EW Lidar module [30] is connected to the system for this series of tests. The Lidar provides a scanning range of $270^{\circ }$ and 30 me with an angular resolution of $0.25^{\circ }$ per step at up to 40 Hz. The Lidar system may be affected by variations in the orientation and movement of a vehicle in moving water with a high wind speed and flow. To address this issue, the Lidar is mounted on a Zhiyun CRANE-M2 gimbal stabilizer to sustain the pitch angle, allowing it to continuously scan horizontally. This can eliminate variations in the Lidar measurements and keep the system’s obstacle detection and avoidance capabilities.

The low-level control and reference tracking system implemented on the frontseat computer uses an onboard digital compass and GPS data to guide the USV along a desired compass bearing and velocity. This is achieved using two PID controllers to calculate the necessary rotational and linear velocity. Meanwhile, the path planner and follower system implemented on the backseat computer, generate a Dubins path based on a commanded mission profile of GPS waypoints and times. The ILOS path follower then uses this path to generate the necessary control output to guide the USV through each waypoint of the mission.

When an obstacle in the environment is sensed by the trained DRL agent (Equation (5)), the agent will provide an action from the action space. The waypoint tracker interprets the DRL agent action into a temporary waypoint $\left(N_{temp},E_{temp}\right)$ which is followed until no obstacle is detected; thus navigating away from the nominal path and avoiding the obstacle. The temporary waypoint $\left(N_{temp},E_{temp}\right)$ used to avoid the obstacle is updated after every prediction of the DRL agent at a rate of 1–2 Hz. The process is described in Algorithm 1.
**Algorithm 1:** DRL Path Augmentation for Obstacle Avoidanceinitialization; mission = [[initialization point], [n waypoints of type (N, E)]]; current_path = path between mission waypoints; 
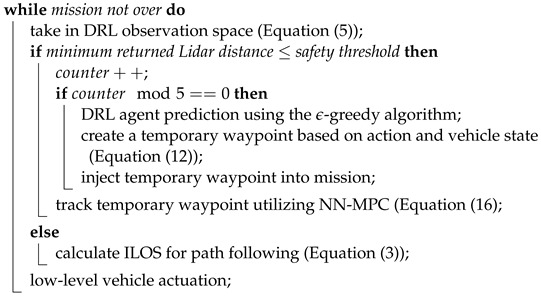


## 3. Results

In this section, we describe the implementation of the proposed method and the results obtained in simulation and real-world experiments. Section 3.1 shows the implementation details of the different simulation environments used for training and testing of the DRL agent. The training results of the DRL agent in environments without wind disturbances are presented in Section 3.2. Section 3.3 presents the training and validation of the DRL agent moving from simulation to field experiments. Finally, Section 3.4 shows the failures caused by wind when NN-MPC is not implemented, then presents the development of the NN-MPC waypoint tracker.

### 3.1. Simulation Environments

**Figure 8:**
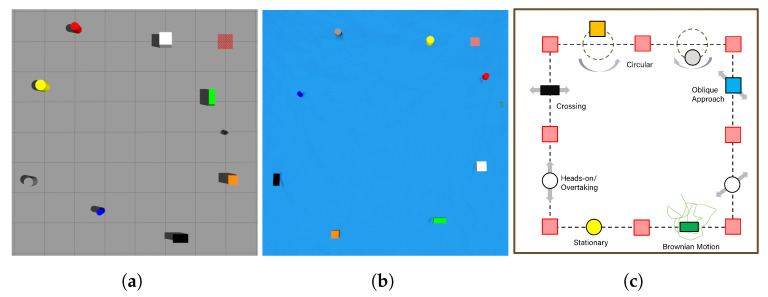
DRL agent training in Gazebo simulation environment and obstacle avoidance mission setup for the DRL agent training. (**a**) UGV training environment. (**b**) USV training environment. (**c**) Obstacle motion types.

The obstacle avoidance strategy aims to train a DRL agent to generate waypoints to avoid obstacles. Two simulated training environments are built in Gazebo, one for the UGV and one for the USV, as illustrated in Figure 8a,b, respectively. These environments do not include environmental disturbances such as wind to accelerate the training. There are eight different waypoints in total. During the training, the robot needs to follow the waypoints 1 to 8 clockwise while avoiding the obstacles, then 7 to 1 counterclockwise and repeat the pattern to better explore the state space. The obstacles have different shapes, sizes, and movement patterns and are placed between mission waypoints. The movement patterns include head-on, crossing, oblique approach, circular, and Brownian motion as shown in Figure 8c. The DQN model is trained during the execution of the waypoint navigation tasks. If a collision occurs, the robot will be reinitialized at the last reached waypoints, the obstacles will be shuffled and another training episode begins.

The simulation is given appropriate sensors for environmental and position feedback, specifically a 2D Lidar, GPS, and IMU. In the ground domain, a TurtleBot is used to train the DRL agent. The course is a 4 m by 4 m square. The obstacle sizes range from 0.3 to 0.5 m and the max obstacle velocity is 0.5 m/s. The Lidar scan range is 3 m. In the water domain, the environment is adapted from the Virtual RobotX (VRX) simulator [31] and the BREAM USV in Figure 6b are used. Additionally, Gazebo “buoyancy” and “USV dynamics” plugins are used to simulate the marine environment and dynamic behavior of the vehicle. Due to the different physical properties of UGV and USV, a larger course and obstacles are set up. The obstacles’ average size is 4 m. The max obstacle velocities are increased to 5 m/s. To sense the obstacles early in the path, the max Lidar scan range is increased to 15 m for the USV.

### 3.2. DQN Network Architecture

**Figure 9:**
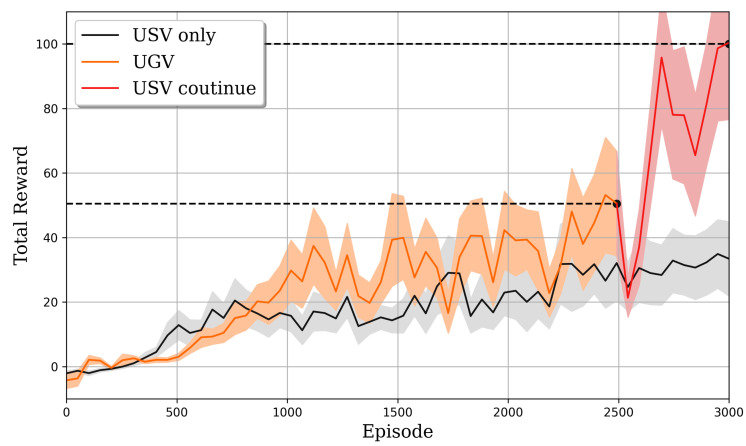
Total reward across training episodes for different training strategies. The black curve shows the reward of a USV being trained for 3000 episodes. The orange and red curve shows the reward of hybrid training. The orange curve is a UGV being trained for 2500 episodes and then the red curve is a USV transferring the policy from the UGV and keeping training for 500 more episodes.

The DQN network was trained on a desktop computer with a GeForce 2080 GPU and an Intel Core i7-8700 CPU. The real-world test is carried out on an NVIDIA Jetson Nano with a 128-core Maxwell GPU and an ARM Cortex A57 CPU. After 2500 training episodes with a learning rate of 0.0001, batch size of 32, and epsilon delay rate of 0.99, the episodic reward reaches approximately 50 when trained on a UGV as shown in Figure 9.

**Figure 10:**
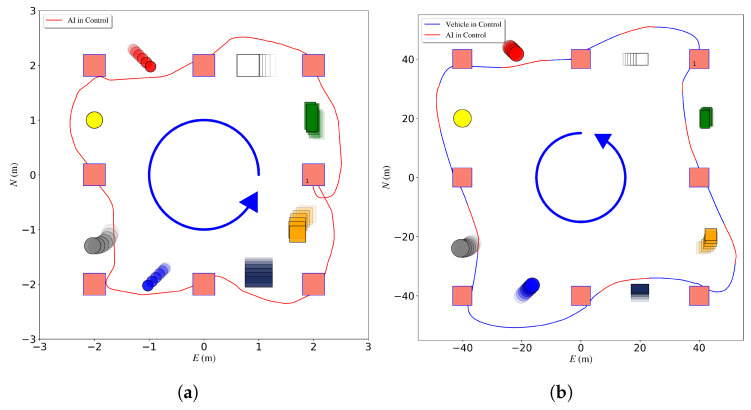
Simulated obstacle avoidance mission showing mission waypoints as red squares, obstacles in different colors, vehicle trajectories in blue when the DRL is activated and trajectories in red when the DRL is activated. The trajectory runs counter-clockwise starting from waypoint 1. (**a**) UGV evaluation in simulation. (**b**) USV evaluation in simulation.

Once the DQN network reaches a reward of approximately 50, the DRL agent can finish the square course with eight waypoints two to three times without collision. The trajectory of finishing one loop is visualized in Figure 10. This model is then loaded on a USV and is trained for 500 episodes in simulation. Then the final reward reaches 100. The total training time is 72 h. Compared to the cross-domain training, the agent only trained on a USV for 3000 can only receive a reward of around 30 with a total training time of more than 100 h.

### 3.3. Cross Domain Training and Validation

**Figure 11:**
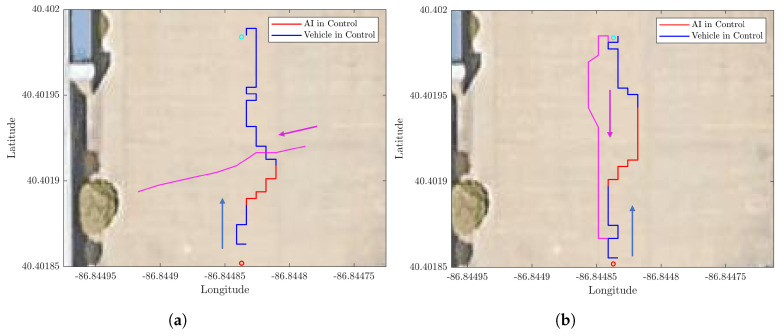
Real-world UGV obstacle avoidance experiment. (**a**) Cross scenario. (**b**) Head-on scenario.

To validate the trained model in real-world scenarios, the model is integrated into the UGV, Figure 7a and tested using a different mission. The course is comprised of two waypoints. The distance between these two waypoints is 20 m. The UGV is required to navigate from one waypoint to another while avoiding one walking human in crossing and head-on scenarios. The robot and the human GPS coordinates were recorded and plotted for the crossing scenario in Figure 11a and the head-on scenario in Figure 11b.

The trajectory has a mean cross-track error of 0.92 with respect to obstacle size. The obstacle size is the average person’s stride length of 0.7 m. The trajectory plots show that the UGV is able to navigate through an environment with dynamic obstacles without collision and find a path to its goals.

To test the DRL agent’s ability in the water domain, evaluation tests have been performed in both simulated and real water environments Virtual RobotX (VRX) simulator [31] and the BREAM USV in Figure 6b are used.

After loading the trained agent, the USV is able to finish the waypoint navigation task without any collisions. The average cross-track error is 0.89 of obstacle size, a maximum deviation from the optimal path of 1.74 of obstacle size. The trajectory is shown in Figure 10b. The obstacle size is 0.4 m.

**Figure 12:**
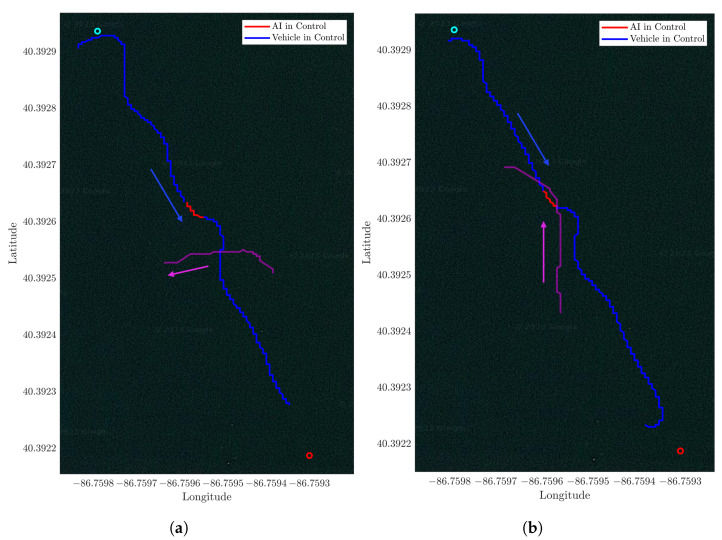
USV avoiding a dynamic obstacle in real-world experiments in crossing and head-on scenario. (**a**) Crossing scenario. (**b**) Head-on scenario.

To verify obstacle avoidance in a real water environment a mission with two waypoints is created. These two points are at a distance of 100 m from each other. The DRL agent tested in real-world experiments does not have any further training. The mission course is run a total of 15 times to avoid a dinghy in heads-on and crossing scenarios, as illustrated in Figure 12a,b, respectively. Only two runs are shown for visual clarity. Throughout the test shown the average cross-track error is 0.93 with respect to the obstacle size, a maximum deviation from the optimal path of 1.72 with respect to the obstacle size. In this test scenario, the obstacle size is the length of the dinghy boat 2.1 m.

### 3.4. NN-MPC Waypoint Tracker

**Figure 13:**
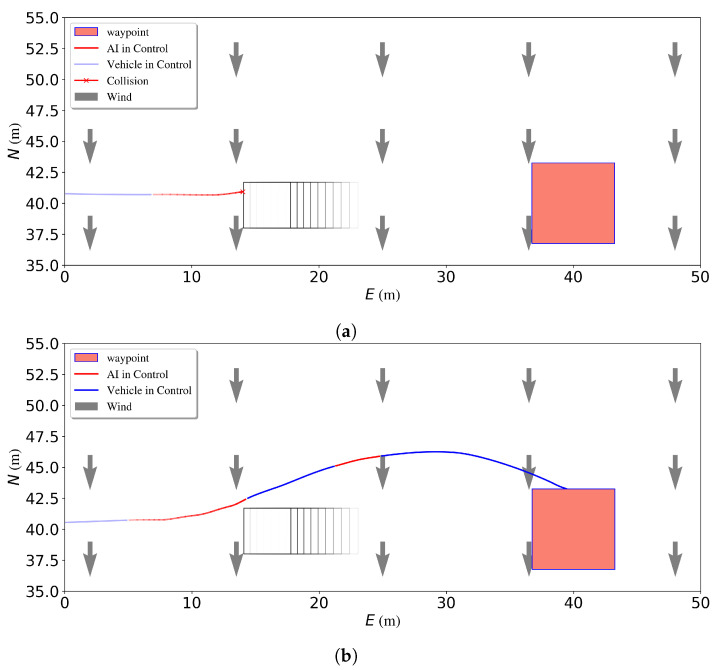
Performance of different waypoint trackers when a USV avoids an obstacle in head-on scenario in simulation. (**a**) Collision happens without using NN-MPC. (**b**) Collision avoidance using NN-MPC.

Due to strong environmental disturbances such as wind, a simple waypoint follower may be unsuccessful in attempting to reach the temporary waypoint generated by the DRL agent even when the correct prediction from the DRL has been made. As shown in Figure 13a, a collision occurs in the head-on scenario when the wind blows from the North while the agent generates a waypoint that requires the vehicle to go against the wind. This failure occurs because the controller is not able to efficiently track the temporary waypoints. As explained in Section 2.4, NN-MPC is used to reject the wind disturbance so that the USV can avoid collision by better following the waypoints.

However, NN-MPC still has some limitations [32,33]. NN-MPC is computationally expensive. Moreover, even with a highly accurate model, there may be uncertainty or disturbances that are not accounted for in the model. This can lead to suboptimal control actions and reduced system performance. To tackle these limitations, we first fine-tune the MPC parameters and simplify the neural network model to find a good trade-off between performance and speed. Next, we use a multi-stage data collecting technique to train the neural network to make sure the control actions generated by NN-MPC are robust to uncertainties and disturbances.

**Figure 14:**
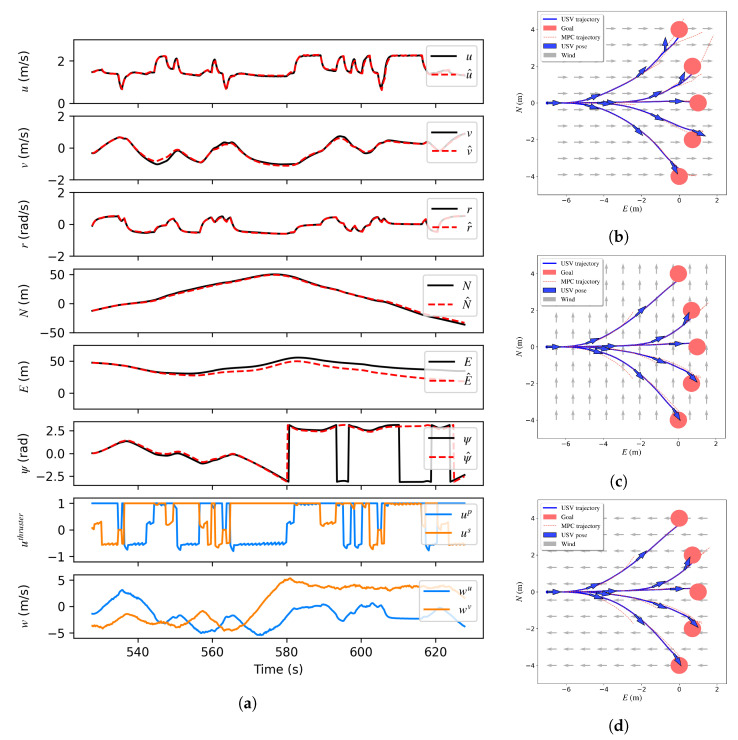
(**a**) is the testing result for the neural network giving initial state and future control inputs and disturbances. The neural network system model is then used by MPC to follow the waypoint under wind disturbance as shown in (**b**–**d**). The blue curves are the USV’s actual trajectories. The red dashed lines are the trajectories generated by MPC. The red circles are the waypoints generated by the DRL agent. (**a**) Measured output *u*,*v*,*r* (black) and model simulation $\hat{u}$,$\hat{v}$,$\hat{r}$ (red) obtained by the trained neural model. (**b**) Along the wind. (**c**) Perpendicular to the wind. (**d**) Against the wind.

A dataset is built to train a neural network to estimate the system dynamics of the BREAM USV. First, the USV is manually driven in the Gazebo simulator to collect data for simple motions such as going straight, making turns, and going backward under different wind disturbances including no wind, constant wind, and varying wind conditions. Then a model is trained with this dataset. Next, the model is used by the MPC in the obstacle avoidance environment to further explore the state space. Throughout this process, more data are collected to augment the initial dataset and further refine the neural network model to make it more robust to uncertainties and disturbances. The network is tested on an unseen test set for around 100 s. The result is shown in Figure 14a. The mean errors of the state estimation are [0.01 m/s, 0.02 m/s, 0.01 rad/s, 1.30 m, 6.62 m, 0.29 rad]. The position errors become large if the prediction time window is too big due to the accumulation of integration errors. However, the step size Δt is 0.2 s and the time window is 10 steps for the MPC thus the NN-based model is accurate enough. The network then is used as a system model by the MPC solver to generate optimal control commands.

The NN-MPC is then tested in simulation to track different waypoints under different wind disturbances. Figure 14b–d show the NN-MPC waypoint tracking performance. Waypoints are created based on Equation (12). In the test, *D* and *d* are 8 m and 0.26 rad. Then the PID waypoint tracker is replaced by the NN-MPC. As is shown in Figure 13b, when the agent generates the same high-level action, the robust NN-MPC tracker is able to reject the disturbances and help the vehicle avoid them.

**Figure 15:**
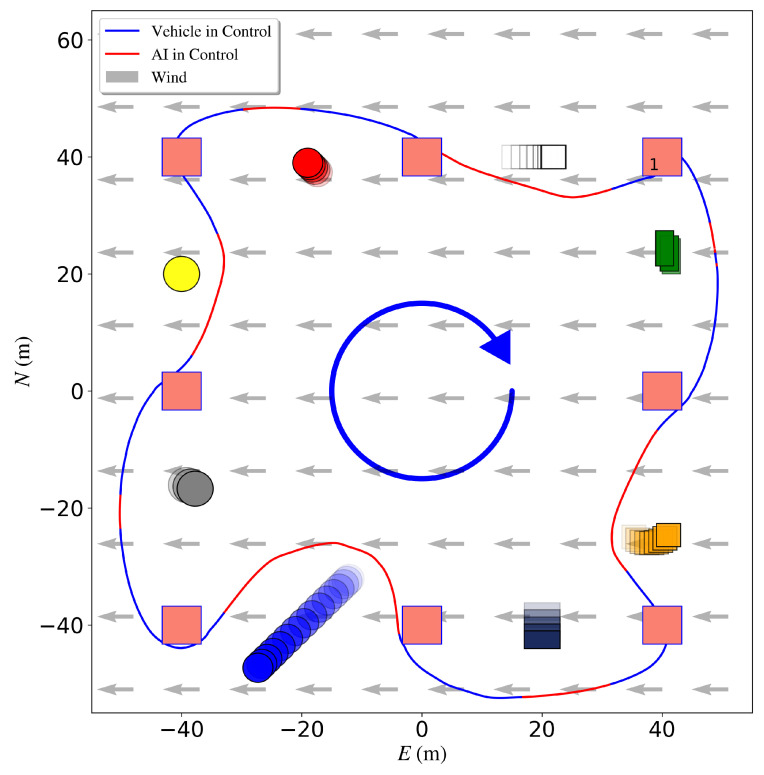
Simulated USV obstacle avoidance mission. Mission waypoints are shown as red squares, obstacles are shown in different colors, DRL agent augmented paths for avoidance in red, and the vehicle trajectory following the mission path in blue. The mission starts from waypoint 1.

Finally, the DRL agent with NN-MPC is evaluated in a similar test as Figure 10b but with wind disturbance from the North. The wind speed is 6 m/s. The trajectory of the whole mission is shown in Figure 15. Even though the agent has not been trained in a windy environment, it is able to finish the mission without collision and the average cross-track error is 1.07 of the obstacle size.

The validations show that the DQN agent is able to rapidly transfer and refine the obstacle avoidance policy from the ground domain to the water domain. With the help of NN-MPC, the agent can achieve good performance under environmental disturbances without further training.

## 4. Conclusions

In this paper, a dynamic obstacle avoidance DRL agent for USVs using cross-domain learning is presented. The generalized obstacle avoidance capability is achieved by utilizing the model free agent that provides path augmentations and the vehicle-dependent tertiary controller that follows the augmented waypoints. Compared to pure water domain training, cross-domain transfer learning decreases the training time by 28% and increases the mean episodic reward from 30 to 100 points. The obstacle avoidance ability of the methodology is validated with a DQN on a UGV and USV simulation as well as on a real UGV and USV. In each case, the DRL agent is able to avoid common dynamic obstacles successfully with a mean path deviation of around 1 with respect to the obstacle size. The results show that the prescribed methodology not only aids in transferring the obstacle avoidance policy from ground to water domains but also helps the sim-to-real transfer. Additionally, NN-MPC is built for the USV as a low-level controller to track temporary waypoints generated by the DRL agent and avoid obstacles in an environment with wind disturbance of 6 m/s, which allows the agent to achieve generalized performance in dynamic environments and can be extended to reject other disturbances such as waves.

Future work on this project is extensive. The model-free DRL agent in this project only avoids one obstacle at a time. However, it may fail when facing a situation in which it needs to navigate around several moving objects. Thus, the agent can be trained in more intricate settings to further improve the policy. The tests will be expanded to validate the proposed methodology on more advanced DRL models such as Actor-Critic and Proximal Policy Optimization (PPO) to generate continuously augmented waypoints and on higher-dimension sensors such as 3-D Lidar and sonar. These more advanced methods and sensors will be used to handle more complex navigational problems. The methodology detailed in this work also shows promise in allowing the training of complex models in the underwater domain through aerial deployment and overall simulation to real-world transfer. However, further investigation is required to verify the feasibility of transitioning between the domains in more complex settings.

## Data Availability

Not applicable.
